# Genetic associations with disease in populations with Indigenous American ancestries

**DOI:** 10.1590/1678-4685-GMB-2023-0024

**Published:** 2024-09-09

**Authors:** Lucas Vicuña

**Affiliations:** 1 University of Chicago, Department of Medicine, Section of Genetic Medicine, Chicago, USA.

**Keywords:** Indigenous Americans, disease risk, GWAS, genetic ancestry, genetic architecture

## Abstract

The genetic architecture of complex diseases affecting populations with Indigenous American ancestries is poorly understood due to their underrepresentation in genomics studies. While most of the genetic diversity associated with disease trait variation is shared among worldwide populations, a fraction of this component is expected to be unique to each continental group, including Indigenous Americans. Here, I describe the current state of knowledge from genome-wide association studies on Indigenous populations, as well as non-Indigenous populations with partial Indigenous ancestries from the American continent, focusing on disease susceptibility and anthropometric traits. While some studies identified risk alleles unique to Indigenous populations, their effects on trait variation are mostly small. I suggest that the associations rendered by many inter-population studies are probably inflated due to the absence of socio-cultural-economic covariates in the association models. I encourage the inclusion of admixed individuals in future GWAS studies to control for inter-ancestry differences in environmental factors. I suggest that some complex diseases might have arisen as *trade-off* costs of adaptations to past evolutionary selective pressures. Finally, I discuss how expanding panels with Indigenous ancestries in GWAS studies is key to accurately assess genetic risk in populations from the American continent, thus decreasing global health disparities.

## Preliminary statement

In this review I describe associations between global ancestry proportions and disease traits, as well as genome-wide associations with anthropometric traits and complex diseases of diverse categories. Even though environmental factors contribute an enormous extent to complex diseases, this review focuses on genetic diversity and its association with disease; thus, I do not discuss environmental causes of disease risk in depth. However, I do emphasize socio-economic factors affecting disease risk when applicable. Also, I focus on associations reported to be unique to populations with Indigenous American genetic ancestries, regardless of their effect sizes on disease traits. For simplicity, in most cases I refer to *genetic ancestries* as *ancestries* and to *significant association* as *association.* It is noteworthy that the overrepresentation of certain Indigenous populations in medical genomic studies can create the false impression that these populations are particularly prone to disease.

Populations with Indigenous American ancestries are a broad group that can be roughly divided into two groups: Indigenous and non-Indigenous populations. Indigenous groups self-identify as *Indigenous* and in general derive all or most of their genetic diversity from ancient North, Central, and South American populations. Non-Indigenous populations in general inhabit the same regions do not self-identify as Indigenous, but derive part of their ancestry from ancient Indigenous populations. For simplicity, I refer to these groups as *Indigenous* and *admixed non-Indigenous* populations, respectively. Although imperfect, these terms are more suitable than US-centric labels such as *Latin Americans* or *Hispanics*, which are used by several studies cited in this review.

## Genetic risk for complex diseases

Heritable genetic variation in humans comprises at least 125 million single nucleotide variants and ~173 thousand structural variants across diverse populations around the world (Byrska-Bishop et al., 2022). However, only ~1 million single nucleotide variants and an undefined number of structural variants have predicted functional consequences (Byrska-Bishop *et al*., 2022), suggesting that they constitute the main substrate for the heritable component of diseases.

In contrast to classic Mendelian *monogenic* diseases, which are caused by a single locus, *complex* diseases are impacted by the sum of many small-effect loci across the genome. The number of these loci, together with their frequencies and effect sizes, constitute the *genetic architecture* of complex diseases (Simons et al., 2018). The genetic architecture of a disease is estimated from its *polygenic score*, a quantity that summarizes the estimated effect of many genetic variants on an individual’s trait. These effects are obtained from *genome-wide association studies* (GWAS), namely, statistical associations between genetic variants across the genome and a trait of interest in a given population. 

GWAS studies have identified hundreds of thousands of genetic variants involved in the genetic architecture of common complex traits and diseases (Welter et al., 2014). Thanks to GWAS studies, we know that most genetic variation associated with disease susceptibility is broadly shared among human populations around the world (Bergström et al., 2020). However, genetic drift (i.e., random changes in allele frequency over time) driven by demographic processes, as well as natural selection and other evolutionary forces, shift allele frequencies in specific populations (Prohaska et al., 2019). Consequently, some risk alleles are common (allele frequency > 0.01) in understudied populations, but rare (allele frequency ⩽ 0.01) in global datasets (Auton et al., 2015; Martin et al., 2017). In addition, there are numerous rare risk alleles that are unique to single or related populations (Auton *et al*., 2015). 

Despite individuals of European ancestries constituting only 14% of the world population (United Nations Report, 2022), two-thirds of GWAS studies have focused on populations with European ancestries (Ju et al., 2022), thus limiting the portability of polygenic scores to other continental populations. For example, polygenic risk scores for schizophrenia derived from Eurocentric data were 50% less accurate in predicting risk for African and East Asian populations (Vilhjálmsson et al., 2015). This underrepresentation has led to health disparities (Martin et al., 2019), inaccuracies in genetic risk assessment, and biased medical recommendations for individuals with non-European ancestries (Wojcik et al., 2019). 

**Figure 1 -:**
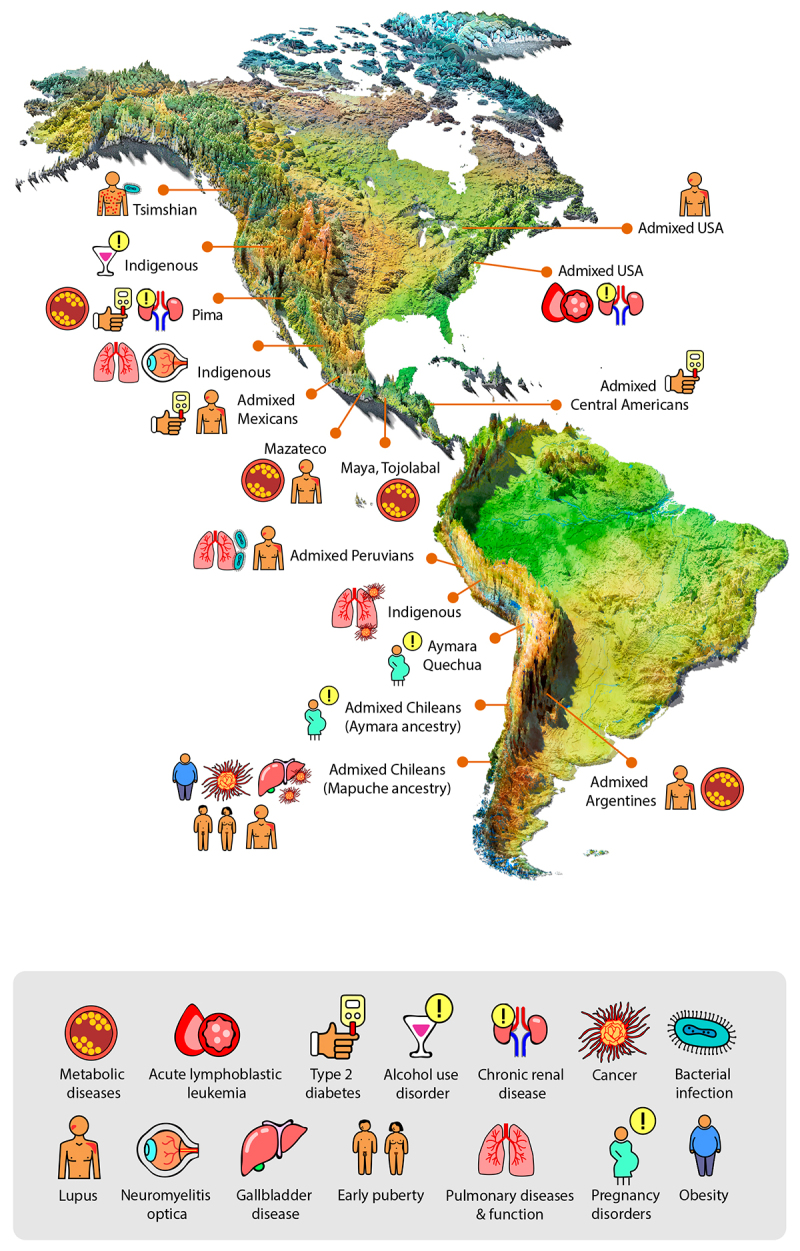
Complex diseases affecting present-day populations with Indigenous ancestries. Map showing diseases or medical conditions (represented by icons) affecting Indigenous and admixed non-Indigenous populations throughout the American continent.

Populations with Indigenous ancestries are particularly underrepresented in GWAS studies (Ju et al., 2022; Kang and Ruderfer, 2020). As of 2021, the estimated number of Indigenous individuals from countries other than the United States and Canada was 57.7 million, that is, 9.8% of the 589.2 million inhabitants of these countries (CRS Report, 2023). In addition, the estimated number of Indigenous individuals in the United States and Canada in 2021 was 4.3 million (US Census Bureau, 2022) and 1.8 million (Canada Census, 2021), respectively. Indigenous and admixed non-Indigenous people represent roughly 0.81% and 7.5% of the world population (United Nations Report, 2022), respectively. However, only 0.03% and 3.85% of GWAS studies have been performed on these populations, respectively (Ju *et al*., 2022). Moreover, to my knowledge there are only a few GWAS performed on Indigenous populations from the United States (Malhotra et al., 2011; Hanson et al., 2014; Brown et al., 2017; Peng et al., 2019; Ramirez-Luzuriaga et al., 2024) and none from Canada. In conclusion, the inclusion of Indigenous and admixed non-Indigenous populations in large-scale GWAS holds significant potential to uncover risk variants specific to Indigenous populations that can impact hundreds of millions of people. Figure 1 depicts the geographic location of populations whose Indigenous genetic diversity has been associated with complex diseases. Table 1 summarizes the genetic factors associated with complex diseases and traits.

**Table 1 -:** Genetic factors affecting diseases and traits in populations with Indigenous ancestries. Populations are sorted from north to south along the American continent.

Population	Region	Disease/Trait	Gene(s)/Ancestry	Reference
Indigenous	American continent	Lower LDL cholesterol	Indigenous ancestries	Acuña-Alonzo et al. (2010) Sohail et al. (2023)
Admixed	USA, Mexico, Peru, Chile, Argentina	Systemic lupus erythematosus	*IRF5/TNPO3*	Alarcón-Riquelme et al. (2016) Langefeld et al. (2017)
Tsimshian	Canada (British Columbia)	Smallpox	*HLA-DQA1*	Lindo et al. (2016)
Indigenous	USA (Arizona)	Pubertal traits	*NUDT3, PACSIN1*	Ramirez-Luzuriaga et al. (2024)
Indigenous	USA (California)	Alcohol use disorders	*PRKG2, FSTL5*	Peng et al. (2019)
Pima	USA	Type 2 diabetes	*DNER*	Hanson et al. (2014)
Pima	USA	Chronic renal disease	*BCL2L11*	Brown et al. (2017)
Admixed	USA	Chronic renal disease	*BCL2L11*	Brown et al. (2017)
Admixed	USA (California)	Acute lymphoblastic leukemia	*PDE4B*, Indigenous ancestries	Yang et al. (2011)
Admixed USA Mexicans	USA	Short stature	Indigenous ancestries	Spear et al. (2020)
Admixed USA Mexicans	USA	High triglyceride & glucose levels (metabolic disorders)	Indigenous ancestries	Spear et al. (2020) Ko et al. (2014)
Admixed USA Mexicans	USA	Type 2 diabetes in obese women	Indigenous ancestries	Hu et al. (2015)
Indigenous	Mexico	Lung function	Indigenous subancestry (West-East cline)	Moreno-Estrada et al. (2014)
Indigenous	Mexico	Neuromyelitis optica	*HLA*	Romero-Hidalgo et al. (2020)
Mayan, Tojolabal	Mexico	Shorter stature than Northern Mexico	Indigenous subancestry	Sohail et al. (2023)
Mayan, Tojolabal	Mexico	Higher triglyceride & glucose levels than Northern Mexico	Indigenous subancestry	Sohail et al. (2023)
Mazateco	Mexico	Systemic lupus erythematosus	*IRF5*	Reddy et al. (2007)
Admixed	Mexico	Lower HDL cholesterol	ABC1*C230, Indigenous ancestries	Spear et al. (2020) Sohail et al. (2023)
Admixed	Mexico, USA	Type 2 diabetes	*SLC16A11*	Williams et al. (2014) Romero-Hidalgo et al. (2020)
Admixed	Puerto Rico	Lung function	*SLIT3*, Indigenous ancestries	Lee et al. (2020)
Admixed	Central America (El Salvador, Guatemala, Honduras, Nicaragua)	Type 2 diabetes	*EPHB1, KY*; Indigenous ancestries	Horimoto et al. (2022)
Admixed	Peru (Lima)	Pulmonary Tuberculosis	*ATP1B3*, Indigenous ancestries; eQTLs	Luo et al. (2019) Asgari et al. (2022) Nathan et al. (2022)
Admixed	Peru (Lima)	Short stature	*FBN1*	Asgari et al. (2020)
Aymara-Quechua-Uro	Peru (Puno)	Preeclampsia	*PROZ, F7, F10*	Nieves-Colón et al. (2022)
Admixed	Chile	Newborn death due to pregnancy disorders	Indigenous subancestry (Aymara-Quechua)	Koenigstein et al. (2021)
Admixed	Chile	Gallbladder disease	*ACBG8*, Indigenous subancestry (Mapuche)	Bustos et al. (2019)
Admixed	Chile	Gallbladder, stomach & esophagus cancers	Indigenous subancestry (Mapuche)	Lorenzo-Bermejo et al. (2017)
Admixed	Chile	Early pubertal onset	Indigenous subancestry (Mapuche)	Vicuña et al. (2021) Vicuña *et al*. (2023)
Admixed	Chile	Pulmonary diseases	Indigenous subancestry (Mapuche)	Lorenzo-Bermejo et al. (2017)
Admixed	Chile, Peru, Brazil, Colombia, Mexico	Nose morphology	*EDAR, PAX3, DCHS2, SUPT3H/RUNX2, GLI3, PAX1*; Mapuche subancestry relative to Central Andean subancestry	Adhikari et al. (2016) Bonfante et al. (2021) Chacón-Duque et al. (2018)

Regarding the genetic risk of particular ancestry groups, including populations with Indigenous ancestries, our current understanding is derived from GWAS studies following three general cohort designs: (i) within-population analyses on a particular ancestry group; (ii) cross-population analyses involving different ancestry groups, whereby variant effect sizes are estimated separately in each group; and (iii) analyses on admixed populations with ancestry from two or more groups, where *global* (i.e., individual) and in some cases *local* (i.e., per variant-wise) genetic ancestries are modeled as covariates. In addition, independent association studies based on one or more of the aforementioned designs can be aggregated in large-scale meta-analyses.

## Categories of complex diseases

### Infectious diseases

On the American continent, infectious diseases such as tuberculosis, influenza and COVID-19 are usually more prevalent in Indigenous groups compared to other ancestries (Rojas, 2007; Tollefson et al., 2013; Soto-Cabezas et al., 2022; Freitas Vaz et al., 2024), largely due to socio-economic disparities (Lee et al., 2023). The role of genetic ancestry in infectious disease risk is unclear, as socio-economic and environmental factors are often unassessed in genetic studies and correlate with genetic ancestry among Indigenous descendants. However, a few studies have found ancestry effects by accounting for socio-demographic factors. For example, among Peruvians from Lima, who in average have a proportion of 80% of Indigenous ancestries, there was a threefold increase in tuberculosis (TB) risk between individuals with the most and least Indigenous ancestries (Asgari et al., 2022). This effect was mostly driven by gene expression regulation, as revealed by ~800 Indigenous-specific expression quantitative trait loci (eQTLs) for TB immune response (Supplementary Table 2 from Asgari *et al*., 2022). Another study from the same group identified a single locus within an enhancer region that possibly alters monocyte function through the action of *ATP1B3* gene (Luo et al., 2019). 

If higher proportions of some Indigenous ancestries have an effect on susceptibility for TB, it is possible that genetic diversity at key immune loci could play a role. For example, human leukocyte antigen (*HLA*) and killer-cell immunoglobulin-like receptors (*KIR*) genes have the lowest genetic diversity among Amazonian Indigenous populations compared to many worldwide populations (de Brito Vargas et al., 2022), which could translate in a less diverse immune repertoire. Arguably, this low genetic diversity might have resulted from lower effective population sizes among Indigenous groups compared to the other continental populations (Wang et al., 2007). 

Overall, additional studies with proper sample sizes and which account for socio-economic status are needed to attest whether or not genetic ancestry has an effect on susceptibility to some infectious diseases. These studies would also be important to identify more infectious disease-associated loci, as well as the distribution of their effects and phenotypic consequences.

### Autoimmune diseases

Systemic lupus erythematosus (SLE) is an autoimmune disease with a high heritability (~66%), according to a twin study (Lawrence et al., 1987). Compared with European ancestries, increases in the proportion of Indigenous ancestries correlate with an earlier SLE onset. However, as SLE morbidity has been shown to correlate with lower socio-economic status, it cannot be ruled out that socio-economic factors also affect SLE onset (Sánchez et al., 2012). Differences in genetic susceptibility for SLE between populations with Indigenous and European ancestries might be explained at least in part by differing allele frequencies at genes involved in SLE risk. One example pertains to *IRF5*, a transcription factor with key roles in the innate immune response (Reddy et al., 2007). A case-control association study on the *IRF5* gene found the strongest association with SLE at the risk allele *rs2070197-C*. This variant allele has a substantially higher frequency in the Indigenous Mazateco from Mexico than in admixed Mexicans, and it is also higher in the latter group than in populations with European ancestries (Reddy *et al*., 2007). Similarly, a GWAS performed on a multi-national cohort with enriched Indigenous ancestries from Argentina, Chile, Peru, Mexico, and the United States captured the strongest hit at a region mapping *IRF5* and its neighboring gene *TNPO3* (Alarcón-Riquelme et al., 2016). Importantly, a large-scale transancestry GWAS replicated the *IRF5*/*TNPO3* signal as the top hit for admixed non-Indigenous individuals, but not for individuals with European nor African ancestries; in these ancestries the top associations were *HLA* variants (Langefeld et al., 2017). These findings further support the hypothesis that inter-population differences in SLE genetic risk are partially due to differences in allele frequencies.

Another autoimmune disease that has been linked to Indigenous ancestries is neuromyelitis optica, a disease of the central nervous system that affects the optic nerve and spinal cord. A case-control GWAS study on admixed Mexicans showed that affected individuals had a higher proportion of Indigenous ancestries than healthy controls. Specifically, *HLA* variants comprised the strongest associated GWAS loci, all of which had increased means of local Indigenous ancestries (Romero-Hidalgo et al., 2020). These observations contribute to the idea that among Indigenous descendants, genetic diversity of the *HLA* region contributes to the development of the disease.

### Metabolic diseases

Increases in proportions of Indigenous ancestries seem to correlate with higher levels of lipid-related traits and diseases, at least in Mexicans (Acuña-Alonzo et al., 2010; Ko et al., 2014; Hu et al., 2015; Huerta-Chagoya et al., 2020; Spear et al., 2020; Sohail et al., 2023). However, as mentioned before, genetic ancestry usually covaries with environmental factors, such as socio-economic status (Sohail *et al*., 2023), making the genetic component difficult to disentangle-occasionally, genetic and environmental factors act in opposite directions, obscuring disease associations altogether. Among admixed Mexicans, higher proportions of Indigenous ancestries associate with higher waist to hip ratio, triglyceride levels, glucose levels (Spear *et al*., 2020), and risk for type 2 diabetes (T2D) among obese women (Hu *et al*., 2015), but also with lower LDL cholesterol levels (colloquially called *bad cholesterol*) (Sohail *et al*., 2023). However, a case-control GWAS for T2D performed in Mexicans and in an admixed non-Indigenous cohort from the United States identified an association for the solute carrier gene *SLC16A11*, at a variant that increases intracellular triglyceride levels. Interestingly, the risk allele has a frequency of 0.5 in Indigenous populations, at least five times higher than in other continental ancestries (Williams et al., 2014). Further, higher Indigenous ancestries are associated with lower HDL cholesterol in 80% of Indigenous populations from the American continent (Acuña-Alonzo *et al*., 2010), thus increasing their collective risk of cardiovascular disease. HDL, usually referred to as *good* cholesterol, is the lipoprotein that removes cholesterol from the blood and transports it back to the liver. This association may be partially explained by a variant in the *ABCA1* transporter gene, which has a key function in the biosynthesis of HDL. The ABC1*C230 allele is found exclusively in Indigenous groups and shows the strongest association with low HDL among Pima from North America’s Southwest and Mayans from Central America (Acuña-Alonzo *et al*., 2010; Hünemeier et al., 2012). However, when all these Indigenous groups are considered together and environmental covariates like socio-cultural factors and diet are included, the genetic association with low HDL does not reach the genome-wide association threshold (Sohail *et al*., 2023). This suggests that the aforementioned environmental factors, and/or possibly an ancient selection event acting at this locus (Hünemeier *et al*., 2012), are responsible for the lack of significant associations. We see a similar interplay between genetics and environment with BMI among admixed Mexicans. *Runs of homozygosity*, which correlate with lower genetic diversity and are more frequent in Indigenous groups, including those from Mexico, are associated with lower BMI (Sohail *et al*., 2023). However, urban environments are associated with higher BMI in admixed Mexicans. We see the same correlation for Indigenous people from Mexico, but only when they live in urban environments (Sohail *et al*., 2023). These findings suggest that if there are genetic risk variants of metabolic diseases among Indigenous groups from Mexico, these variants need to interact with the urban environment (e.g. diet, sedentarism) for obesity to be triggered (Sohail *et al*., 2023). Taken together, these studies suggest that while genetic factors might contribute to disease risk, environmental factors have a profound effect on cardiovascular disease in Indigenous populations. 

In addition to lipid dysregulation, T2D is a significant concern among many Indigenous populations. While modern Western dietary trends likely have a significant influence on the wide-spread development of T2D, recent studies have also uncovered genetic factors unique to Indigenous populations that may contribute to susceptibility. In a cohort of Central American heritage (El Salvador, Guatemala, Honduras, Nicaragua) from the United States, an *admixture mapping* study -associations between a trait and the per SNP-wise mean local ancestry along the genome- identified an haplotype block harboring the multifunctional *KY* and *EPHB1* genes, where Indigenous ancestries conferred increased risk for T2D, after accounting for socio-demographic variables (Horimoto et al., 2022). The Indigenous Pima have one of the highest incidences of T2D in the world (Schulz and Chaudhari, 2015), and part of this risk might be affected by genetic loci, as suggested by a GWAS study, which identified a *DNER* gene risk variant associated with T2D. *DNER* encodes a growth factor that mediates signaling in the insulin-secreting pancreatic ß-cells (Hanson et al., 2014). A related GWAS for BMI on the same population identified several associations, but none reached the genome-wide significance threshold (Malhotra et al., 2011). These two GWAS were not adjusted for socio-economic factors. Thus, while the results suggest that Indigenous populations may be more genetically susceptible to the negative effects of the modern Western diet on metabolic function, this hypothesis needs to be tested by rigorously assessing the contribution of environmental factors. 

### Cancer

Increased proportions of Indigenous ancestries have been associated with an increased incidence of certain cancers. A genome-wide study performed in a cohort from California, showed that higher individual proportions of Indigenous ancestries, as well as a higher mean of local Indigenous ancestries at *PDE4B* variants, were associated with higher recurrence of acute lymphoblastic leukemia. However, as noted by the authors, the effect of environmental, socio-economic, and dietary factors in these associations cannot be ruled out (Yang et al., 2011)**.**

Indigenous subancestries might have opposite effects on some kinds of cancers. A genetic epidemiology study based on aggregated data, performed associations between regional proportions of Indigenous subancestries in admixed Chileans and their mortality rates due to several cancers, adjusting for individual socio-economic variables (Lorenzo-Bermejo et al., 2017). The most striking finding was the strong positive association between Mapuche subancestry (Northern Patagonia lowlands) and mortality risk for gallbladder cancer; specifically, a 3.7% increased mortality per 1% increase in Mapuche subancestry. Nevertheless, hitherto no variant has achieved a genome-wide association with gallbladder cancer among Mapuche ancestry-bearing populations. However, in admixed Chileans a *ACBG8* gene variant is associated with gallbladder disease, which is the main risk factor for gallbladder cancer (Bustos et al., 2019). The same study from Lorenzo-Bermejo *et al.* (2017) reported that while Mapuche subancestry is associated with increased mortality rates due to esophagus and stomach cancer, Aymara subancestry (Central Andes highlands) has a protective effect for these cancer types. In addition, the mortality rate due to skin, bladder, larynx, bronchus, and lung cancers decreases with augmented Mapuche subancestry proportions. While the findings on subancestry differences in mortality rates are promising for health policies targeting particular ethnic groups, they need to be validated using individual paired genetic-phenotypic data.

### Pulmonary diseases

As with cancer, Indigenous subancestries also seem to have contrasting effects on pulmonary disease outcomes. While Mapuche subancestry among admixed Chileans is associated with increased mortality due to asthma, pneumonia, and chronic lower respiratory diseases, Aymara subancestry appears to have a protective effect (Lorenzo-Bermejo et al., 2017). It is possible that positive selection among the Aymara for physiological traits enabling life at the high altitudes of the Andes Mountains, such as hypoxia and cardiovascular function (Crawford et al., 2017), might have contributed to these differences. Among Mexicans, Indigenous subancestry from the western states of the country (e.g., Sonora) associates with a 7% change in lung function (i.e., severity of obstructive lung diseases) compared with the eastern states (e.g., Yucatán) (Moreno-Estrada et al., 2014). In a cohort of ~5,500 admixed individuals with African, European, and Indigenous ancestries recruited in the United States, Indigenous ancestries associated with lower odds of asthma, after adjusting for early life exposures, air pollution and socioeconomic status (Pino-Yanes et al., 2015). An admixture mapping study performed on admixed Puerto Ricans identified a chromosomal region where each Indigenous allele was associated with an increase in lung function. The lead SNP mapped the tumor suppression *SLIT3* gene (Lee et al., 2020). It remains to be determined which genetic and non-genetic variables contribute the most to such fine-scale differences in lung function among Indigenous subancestries.

### Renal diseases

Chronic kidney disease (CKD) is a serious condition characterized by kidney damage and impaired ability to filter waste from the blood. In Central American countries with high Indigenous heritage like Nicaragua, Costa Rica, El Salvador, and Guatemala, CKD is highly prevalent (Horimoto et al., 2022). Also, in the United States the prevalence of CKD among Indigenous populations is double that of other ethnic groups. An admixture mapping study of albuminuria-increased albumin excretion, which is a sign of kidney damage-performed on ~12,000 admixed individuals with sociodemographic assessment, identified two signals enriched in Indigenous ancestries associated with increased urine albumin excretion. One of them harbors a variant mapping the *BCL2L11* gene, which has putative roles in kidney function and disease. Interestingly, the associated allele has a frequency higher than 0.5 among the Indigenous Pima but is non-existent in European and African populations (Brown et al., 2017). A similar admixture mapping study from the same group performed on a cohort of Central American origin identified a haplotype spanning the *RGS6* gene, whereby the allele enriched in Indigenous ancestries has protective effects for albuminuria over the allele enriched in European ancestries (Horimoto *et al*., 2022). These results suggest that at least part of the genetic diversity of Indigenous populations underlies specific effects on susceptibility or protection to kidney diseases.

### Neuropsychiatric disorders

Indigenous ancestries appear to confer a reduced risk of developing Alzheimer’s disease, as suggested by genetic studies in admixed Brazilians (Benedet et al., 2012), Colombians (Moreno et al., 2017), and admixed Caribbean individuals (Horimoto et al., 2021). An admixture mapping study in the latter population found that an excess of Indigenous ancestries at a region harboring five protein-coding genes, associated with a protective effect against Alzheimer’s disease risk. Within that region, one variant allele mapping the *DUBR* gene was associated with a reduced risk for Alzheimer’s disease in individuals with European ancestries (Horimoto *et al*., 2021). *DUBR* encodes a long noncoding RNA (lncRNA) implicated in brain development and function (Huang et al., 2022). 

Alcohol dependence has a relevant genetic component, with an estimated heritability of 50%, according to twin and adoption studies (Verhulst et al., 2015). Epidemiological data suggests that some Indigenous groups are more prone to alcohol use disorders than other populations (Ehlers and Gizer, 2013). As of 2005, alcohol dependence rates were 5-6 times higher among Indigenous people from the United States compared to other United States ethnic populations (Ehlers and Wilhelmsen 2005). To (partially) explain these observations, Ehlers and Gizer (2013), hypothesized a scenario of gene-by-environment interaction, whereby the disorder’s morbidity is increased by the exposure of genetic risk variants to certain environments, such as low socio-economic status and historical trauma, that are more common among Indigenous populations than in other populations. 

A GWAS study for alcohol dependence performed on an Indigenous cohort from California, as well as on a cohort of European ancestries revealed a few associations. While common variants in the uncharacterized *FSTL5* gene, associated with alcohol consumption in the population with European ancestries, rare variants in *FSTL5* and *PRKG2* genes associated with alcohol-related life events and with affective symptoms when cutting down alcohol only in the Indigenous group (Peng et al., 2019). Interestingly, *PRKG2* has been associated with obesity-related traits in diverse worldwide populations (Peng *et al*., 2019), suggesting that variants that favor food intake might favor alcohol consumption as well (Ehlers and Gizer, 2013). While these results are promising, the effect of specific variants need to be formally tested in an admixed cohort, controlling for environmental differences between ancestries.

### Pregnancy disorders

Preeclampsia is a medical pregnancy condition that causes 40% of all premature births, and is characterized by high blood pressure as well as signs of liver or kidney damage. The risk for preeclampsia increases in high altitude regions for all ancestries. However, women from Puno-located ~3,800m above sea level in the Peruvian Andes and inhabited by Quechua, Aymara, and Uro Indigenous populations-exhibit one of the highest incidences of preeclampsia in the world, suggesting the susceptibility may have a genetic component in this population. A parent-offspring trio GWAS study among Puno residents found that common genetic variants mapping the clotting factor genes *PROZ*, *F7*, and *F10* in the fetal genome increase susceptibility to preeclampsia among these women (Nieves-Colón et al., 2022). Future studies will help understand whether genetic diversity also underlies risk for preeclampsia in other high-altitude populations worldwide.

Indigenous populations show the lowest genetic diversity worldwide (Wang et al., 2007), and some of them exhibit large runs of homozygosity (Koenigstein et al., 2021), which may result in a higher incidence of birth defects and other diseases. A genetic epidemiology study performed on admixed Chileans, found that runs of homozygosity and Aymara subancestry are associated with increased risk of child mortality due to natural intracranial hemorrhage of fetuses and newborns as well as to related gestation disorders (Koenigstein *et al*., 2021), after correcting for several socio-economic/education variables. However, this study did not use paired genetic-phenotypic data. It remains to be determined whether or not these disease outcomes are similar in other worldwide populations with similar inbreeding levels.

## Anthropometric traits

Stature is a polygenic trait explained by the contribution of thousands of variants (Yengo et al., 2022), each exerting small effect sizes. In the population of Lima, Peru, which as mentioned before has on average 80% Indigenous ancestry and is among the world’s shortest populations, 700 variants explain 7% of the variance in height (Asgari et al., 2020). However, in this population, a single missense variant allele in the *FBN1* gene accounts for 1% of the total height. Hence, one person carrying a single copy of that allele is 2.2 cm shorter on average than people carrying the alternative allele (Asgari *et al*., 2020). *FBN1* encodes an extracellular matrix protein fibrillin-1, which provides mechanical stability to tissues. While candidate genes were not sought, another study found the composition of Indigenous ancestries may have an effect on height: subancestry from Central America (Oaxaca, Maya) is associated with a shorter stature relative to subancestry from Northern Mexico (Huichol, Tarahumara) (Sohail et al., 2023). This is another case where quantitative genetics analyses can detect subtle effects of Indigenous subancestries over a phenotype.

When comparing the effect of ancestry on facial traits in admixed populations from Brazil, Chile, Colombia, Mexico, and Peru, the strongest correlation (opposite effects in Indigenous vs. European ancestries) was found for a measure of nose position (Bonfante et al., 2021). A related GWAS performed in the same cohort identified six SNPs associated with nose shape, mapping the genes *EDAR*, *PAX3*, *DCHS2*, *SUPT3H/RUNX2*, *GLI3,* and *PAX1* to the trait (Adhikari et al., 2016). In a further study on the same sample it was found that Mapuche subancestry strongly associates with a less protruded nose and broader nose-tip angle when contrasted with Central Andean ancestries (Quechua, Colla, and Aymara populations pooled together). Further, SNP alleles associated with these features show differences in their frequencies between these subancestries (Chacón-Duque et al., 2018).

Abnormal pubertal growth is associated with adult risk for cancer, diabetes and cardiometabolic disorders. Pubertal growth variability has a strong genetic component. In an Indigenous population from Arizona, it was found that the heritability of pubertal traits is between 25% and 71% (Ramirez-Luzuriaga et al., 2024). Also, genetic ancestry underlies variation in pubertal traits. Two studies based on the same admixed longitudinal pediatric cohort, quantified how growth traits differ in predicted individuals with 100% Mapuche versus 100% European ancestries. The first study (Vicuña et al., 2021) analyzed the peak height velocity, which is the period where maximum rate of growth occurs during puberty. The authors predicted that the age at peak height velocity is 0.7 years earlier in adolescents with exclusively Mapuche ancestry versus exclusively European ancestries. However, the study did not correct for individual socio-economic status. The second study, which did account for maternal education level, a proxy for socio-economic status, analyzed child growth, by focusing on longitudinal BMI (Vicuña *et al*., 2023). BMI was higher in children with exclusively Mapuche ancestry at all ages older than 6 years old when compared with children with exclusively European ancestries. Further, the age at adiposity rebound (Age-AR)-the age when the minimal BMI is reached during childhood growth-was lower by 1.9 years, and the BMI at Age-AR was higher by 1.2 kg/m2 in children with exclusively Mapuche ancestry versus exclusively European ancestries. These results suggest that Mapuche children have an earlier pubertal onset than children with European ancestries, which can be explained in part by differences in genetic diversity. A recent GWAS study performed on an Indigenous population from Arizona, identified six variants associated with peak height velocity and two with the duration of growth spurt (Ramirez-Luzuriaga *et al*., 2024). The associated genes include *NUDT3* and *PACSIN1*, whose role in puberty is unknown. However, since there are only a few longitudinal studies on puberty, it cannot be attested if these associations are specific to Indigenous populations. 

## Evolutionary causes of genetic risk among Indigenous populations

**Figure 2 -:**
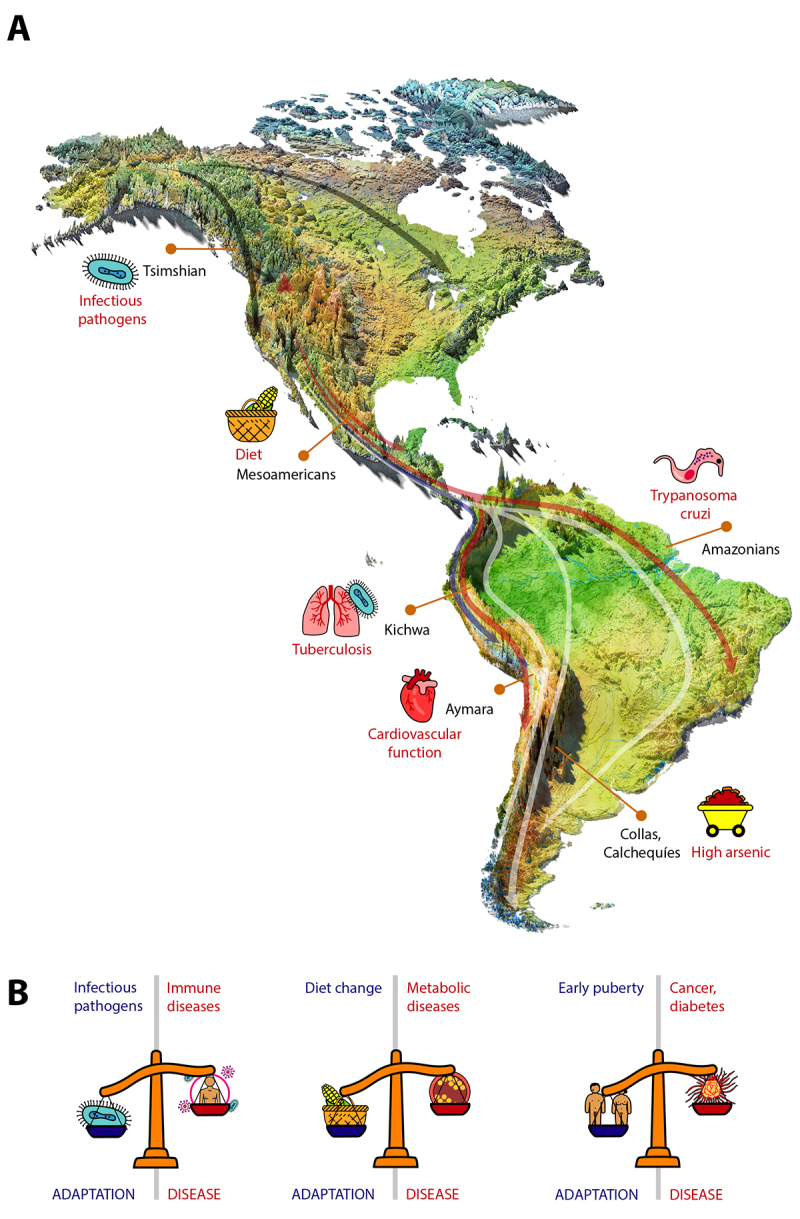
Adaptations experienced by ancient Indigenous groups during the peopling of the American continent. **A.** Representative examples of adaptations experienced by some populations. Arrows represent approximate migration routes of ancient Indigenous populations and arrow colors represent different lineages. Adapted from Posth et al. (2018). **B**. Hypotheses for *trade-offs* between past adaptations leading to diseases in the present. Scales represent the trade-offs between present day diseases and past adaptations.

The first Indigenous Americans entered the North American continent no earlier than 23,000 years ago (Raghavan et al., 2015). Within a few millennia they reached virtually all corners of North, South, and Central America (Figure 2A ). During their journey, they experienced complex demographic events including founder events and population admixture. Also, since arriving in the American continent, the genomes of Indigenous populations have been shaped by their local environments. This began in the ancestors of all Indigenous people, leading to shared signatures of selection across the continent. For example, variant alleles of Denisovan origin in the immune-related *MUC19* gene helped ancient Indigenous populations to adapt to local selective pressures (Villanea et al., 2023). Populations then adapted to their local environments, including selection to high levels of inorganic arsenic in water among Andean populations from Argentina (Collas, Calchequíes) (Schlebusch et al., 2015); cardiovascular function among high-altitude Aymara from Bolivia (Crawford et al., 2017); TB infection among Ecuadorian highland populations (Joseph et al., 2023); parasitic infections among Amazonian groups from Brazil (Couto-Silva et al., 2023); as well as changing diets among ancient Mesoamericans (Hünemeier et al., 2012). Selection continued in response to changing environmental conditions. For example, the introduction of European pathogens likely imposed strong selection across the American continent, causing genetic adaptation in Indigenous populations (Lindo et al., 2016) (Figure 2A ). Moreover, assortative mating conditioned on ancestry-related traits left footprints in the genomes of admixed populations from the American continent (Mas-Sandoval et al., 2023). The cumulative effects of these selective events, genetic drift, as well as genetic forces such as mutation, recombination, and gene conversion, have led to broad differences in disease susceptibility across the American continent, as well as between Indigenous descendants and individuals from other parts of the world.

The precise evolutionary events leading to genetic risk are unknown for most diseases, including those affecting Indigenous populations. However, there are a few instances where these events have been inferred and contextualized, mostly as *trade-offs* between fitness advantages conferred by positive selection acting over target traits in ancestral groups, and fitness costs associated with increased genetic risk at causal, linked, and *pleiotropic loci* (i.e., loci affecting many traits simultaneously) (Figure 2B ). 

For example, selection on the *HLA* locus appears to have favored *HLA-DQA1* among the Indigenous Tsimshian from the Canadian Pacific Northwest before the encounter with Europeans. *HLA-DQA1* alleles were fixed in pre-contact Tsimshian as a result of positive selection, most likely to pathogens endemic to that region. However, population genetics analyses showed that after the introduction of smallpox from Europe, some of these alleles drastically decreased in frequency (as low as 0.37) as a result of negative selection (Lindo et al., 2016). Thus, this example highlights how an increased genetic risk for infectious diseases among Indigenous populations might result from prior positive selection acting on immune variants during environmental (i.e., pathogenic) change (Figure 2B ). 

*Trade-off* hypotheses posit adaptations to famine at genes involved in lipid metabolism among ancient Indigenous populations at the cost of increased risk for dyslipidemias in modern descendants (Ko et al., 2014) [see Neel’s thrifty gene hypothesis (Neel 1962)] (Figure 2B ). Some Indigenous groups from North America seem to have a higher general risk to obesity and lipid-related disorders (i.e., dyslipidemias) than other populations (Breathett et al., 2020; Huerta-Chagoya et al., 2020). Interestingly, admixed Mexicans show Indigenous-specific positive selection signals at a haplotype region harboring the salt-inducible kinase 3 (*SIK3)* gene (Ko *et al*., 2014). The selected *SIK3* haplotype is associated with high triglyceride levels in Mexicans, but not in a European population, as suggested by an allele frequency of less than 0.01 in the latter group. Similarly, evidence suggests that the Indigenous-specific ABC1*C230 allele, which favors the retention of intracellular cholesterol, experienced a selection event in an ancient Indigenous population (Acuña-Alonzo et al., 2010; Hünemeier et al., 2012).

There are other hypotheses of putative *trade-offs* involving food metabolism. A risk variant allele at the gene *SLC16A11* that increases T2D risk in Indigenous populations, introgressed into modern humans via admixture with Neanderthals (SIGMA Type 2 Diabetes Consortium *et al*., 2014). However, it is unknown whether that risk allele conferred selective advantages to the Neanderthal ancestors or human ancestors of present-day Indigenous populations. Another hypothesis posits a higher susceptibility for eating behavior among Indigenous populations, which would be pleiotropically linked with a higher genetic susceptibility to alcohol abuse disorders (Ehlers and Gizer, 2013). This effect would be mediated by variants involved in both traits, such as *PRKG2* variants (see GWAS findings on *PRKG2* mentioned above) (Peng et al., 2019). However, this hypothesis is only supported by indirect evidence.

*Trade-offs* might also exist between selective advantages provided by early pubertal onset and diseases occurring later in life (Figure 2B ). In pygmy groups, early pubertal onset might be favored to achieve higher reproductive rates in conditions of short lifespans and resource availability (Migliano et al., 2007), and positive selection favoring early pubertal onset might play a role here. Interestingly, the Indigenous Hiwi from northern South America are well known for their significantly higher pre-pubertal growth and earlier pubertal onset compared to other groups (Walker et al., 2006). Also, they exhibit short lifespans (e.g., as of 2006, life expectancy at age 15 was 36 years). On the other hand, early puberty can exacerbate disease risk for several diseases in adulthood, including breast and endometrial cancer, T2D, cardiovascular diseases, and psychopathologies (Graber, 2013); (Day et al., 2015; 2017). Even though there is no epidemiological data comparing early puberty-triggered adulthood diseases between the Hiwi and other continental groups, there is evidence for such diseases in other Indigenous groups. For example, among Indigenous males from Arizona, an earlier and accelerated pubertal onset was associated with a higher risk for T2D (Ramirez-Luzuriaga et al., 2023). Also, two genetic studies mentioned earlier showed that Mapuche subancestry leads to an earlier pubertal onset compared to European ancestries (Vicuña et al., 2023, 2021); however, it is not known whether this is the result of positive selection or drift, nor whether this phenomenon has been linked to adult diseases among them. In summary, it is possible that some diseases among Indigenous groups arise as a consequence of early puberty, resulting from an adaptation to short life spans; however, this hypothesis is speculative and would need to be tested.

## Future directions

Many of the disease risk variants have been identified via GWAS based on relatively small cohorts genotyped on SNP arrays, which capture a modest fraction of the genetic architecture of complex diseases. Hence, bigger sample sizes and deeper sequence coverage are needed to estimate the effect of common variants, which should be independent of the population (Hou et al., 2023), as well as to capture the effect of rare variants unique to Indigenous populations. Some initiatives are addressing these gaps through the assembly of relatively large cohorts and/or biobanks, such as Maule Cohort in Chile (MAUCO) (Ferreccio et al., 2020), Estudo Longitudinal de Saúde do Adulto (ELSA) in Brazil (Schmidt et al., 2015), and the Mexican Biobank project (Sohail et al., 2023). Association studies in these populations promise to identify variants associated with disease in Indigenous populations. 

However, association studies are only one piece to identifying causal genetic variants involved in complex diseases that affect populations with Indigenous ancestries. Mapping of molecular quantitative trait loci (mQTLs) holds enormous potential for disentangling the *functional architecture* of these diseases. Thanks to QTL mapping we know that, in addition to ~10% of GWAS hits mapping variants producing changes in amino acids (Hindorff et al., 2009), ~40% of GWAS hits are associated with the expression (eQTLs) and splicing (sQTLs) levels of particular genes (Mu et al., 2021). An additional ~25% GWAS hits mediate epigenetic regulation through their effects on chromatin accessibility (caQTLs) (Zepeng Mu, unpublished data through personal communication), leaving 25% of GWAS hits uncharacterized. 

Fully understanding the causal variants and biological pathways involved in complex diseases affecting Indigenous populations also requires in depth functional characterization using experimental models. For example, expression regulation of the *FTO* gene, a classic gene involved in obesity risk, was thoroughly characterized in adipocytes and brain neurons using functional genomics approaches, leading to key conclusions about pleiotropy in the context of obesity (Sobreira et al., 2021). 

More generally, the implementation of high throughput methods, such as single-cell sequencing and genome editing, is key to identifying genetic variants likely to be functionally important, such as those underlying molecular QTLs. This is particularly relevant to capture inter-population differences in disease risk, given that most of these differences are expected to arise as a result of slight differences in gene regulation across multiple tissues (Watanabe et al., 2019). 

Finally, and maybe most importantly, genetic research involving Indigenous populations raises significant ethical considerations due to historical injustices, cultural sensitivities, and the potential for exploitation (Claw et al., 2018). Thus, fostering ethical partnerships and engagement with Indigenous communities is key to achieve a higher representation of Indigenous populations in genomic studies, which in turn has the potential to reduce the risk for certain diseases for which they are disproportionately susceptible (Claw *et al*., 2018). 

## Conclusions

The genetic architecture of diseases is poorly understood in populations with Indigenous ancestries due to their underrepresentation in medical genomic studies. While most of the genetic architecture of complex diseases is expected to be shared among all human populations, a smaller but relevant fraction is expected to vary among continental populations, including Indigenous populations. Increases in Indigenous genetic ancestries are associated with higher or lower susceptibility for certain diseases. However, in some cases Indigenous subancestries might also have contrasting effects on particular diseases. Indigenous ancestries covary with environmental factors such as socio-economic status, frequently leading to wrong conclusions about genetic risk to complex diseases. The interaction of risk variants with an unfavorable environment (e.g., unhealthy diet) is key for triggering certain diseases (e.g., metabolic disorders) among Indigenous populations. Several findings on higher risk for complex diseases among Indigenous groups are based on cross-population comparisons, mostly with populations with European ancestries. However, it is likely that a substantial fraction of such inter-population risk differences is inflated because of uncontrolled environmental variables and differences in the exact phenotypes assessed across groups (Hou et al., 2023). Future genetic association studies that compare effects across Indigenous and non-Indigenous local ancestries within admixed individuals are needed to reassess many GWAS findings. 
